# Клиническая и молекулярно-генетическая характеристика 3 семейных случаев гонадотропинзависимого преждевременного полового развития, обусловленного мутациями в гене <i>MKRN3</i>

**DOI:** 10.14341/probl12745

**Published:** 2021-05-11

**Authors:** Н. А. Зубкова, А. А. Колодкина, Н. А. Макрецкая, П. Л. Окороков, Т. В. Погода, Е. В. Васильев, В. М. Петров, А. Н. Тюльпаков

**Affiliations:** Национальный медицинский исследовательский центр эндокринологии; Национальный медицинский исследовательский центр эндокринологии; Национальный медицинский исследовательский центр эндокринологии; Национальный медицинский исследовательский центр эндокринологии; Национальный медицинский исследовательский центр эндокринологии; Национальный медицинский исследовательский центр эндокринологии; Национальный медицинский исследовательский центр эндокринологии; Национальный медицинский исследовательский центр эндокринологии; Медико-генетический научный центр им. академика Н.П. Бочкова

**Keywords:** <i>MKRN3</i>, гонадотропинзависимое преждевременное половое развитие, семейная форма

## Abstract

Гонадотропинзависимое (центральное) преждевременное половое развитие (ППР) обусловлено ранней (до 8 лет у девочек и 9 лет у мальчиков) активацией центрального звена гипоталамо-гипофизарно-гонадной системы. Повышение секреции половых стероидов гонадами при данной форме является следствием стимуляции половых желез гонадотропными гормонами гипофиза. В отсутствие аномалий центральной нервной системы центральное ППР классифицируется как идиопатическое и в ряде случаев является наследственным. Инактивирующие мутации в гене MKRN3 являются наиболее частой и известной причиной семейных случаев ППР по сравнению со спорадическими. В настоящей работе впервые в Российской Федерации представлено описание 3 семейных случаев гонадотропинзависимого ППР, обусловленного ранее не описанными мутациями в гене MKRN3, выявленными методом NGS.

## Abstract (en)

Gonadotropin-dependent precocious puberty (central) is a condition resulting from the early (up to 8 years in girls and 9 years in boys) reactivation of the hypothalamic-pituitary-gonadal axis. An increase in the secretion of sex steroids by the gonads in this form is a consequence of the stimulation of the sex glands by gonadotropic hormones of the pituitary gland. In the absence of central nervous system abnormalities, CPP is classified as idiopathic and as familial in some cases, emphasizing the genetic origin of this disorder. Loss-of-function mutations in Makorin Ring Finger Protein 3 (MKRN3) are the most common identified genetic cause of central precocious puberty compared to sporadic cases. In the present study we performed the first descrition of 3 family cases of central precocious puberty duo to novel MKRN3 gene mutation detected by NGS in the Russian Federation.

## АКТУАЛЬНОСТЬ

Центральное (гонадотропинзависимое) преждевременное половое развитие (ППР) обусловлено ранней реактивацией гипоталамо-гипофизарно-гонадной оси и клинически проявляется развитием вторичных половых признаков в возрасте до 8 лет у девочек и 9 лет у мальчиков. Время начала полового созревания определяют сложные взаимодействия между генетическими, алиментарными, экологическими и социально-экономическими факторами [1][2].

Причина 90% случаев ППР у девочек и 25–60% у мальчиков остается неизвестной, в связи с чем принято классифицировать его как идиопатическое ППР. В редких случаях центральное ППР обусловлено поражениями центральной нервной системы (опухоли хиазмально-селлярной области, арахноидальные кисты, травмы, гидроцефалия) [3][4]. Наряду с этим роль генетических факторов убедительно показана в популяционных исследованиях и проиллюстрирована аналогичным возрастом менархе у матерей и дочерей, а также монозиготных близнецов [5][6]. Семейный характер ППР, в отсутствие аномалий центральной нервной системы, позволяет предположить моногенный генез заболевания. К настоящему моменту известно пять генов (KISS1, KISS1R, MKRN3, DLK1, PROKR2), мутации в которых ассоциированы с центральным ППР [7]. Мутации в гене MKRN3 признаны наиболее распространенной причиной моногенных случаев ППР, достигая 33–46% среди семейных вариантов и 0,4–5% — среди спорадических случаев [8][9].

В настоящей работе нами впервые в Российской Федерации приводится описание 3 семейных случаев гонадотропинзависимого ППР у пациентов с доказанными, ранее не описанными дефектами гена MKRN3.

## ОПИСАНИЕ КЛИНИЧЕСКИХ СЛУЧАЕВ

В исследование включены 3 семьи с семейными случаями ППР центрального генеза. В семье 1 гонадотропинзависимое ППР установлено у единоутробных сестер (пробанды 1.1 и 1.2) и их двоюродной сестры по линии отца (1.3), у бабушки по линии отца менархе в 10 лет (рис. 1).

В семье 2 диагноз установлен у двух сестер (пробанды 2.1 и 2.2) из дихориальной диамниотической двойни. У бабушки по линии отца менархе в 10 лет (рис. 2).

В семье 3, наряду с пробандом (девочка 3.1) с ППР, раннее менархе (9 лет) диагностировано у тети по линии отца, данных о возрасте начала пубертата у отца нет (рис. 3).

**Figure:**
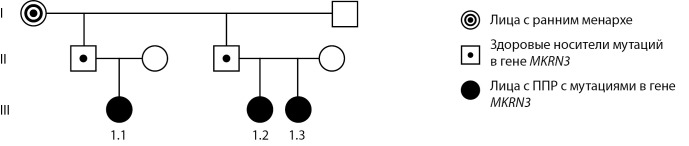
Рисунок 1. Родословная семьи 1.

**Figure:**
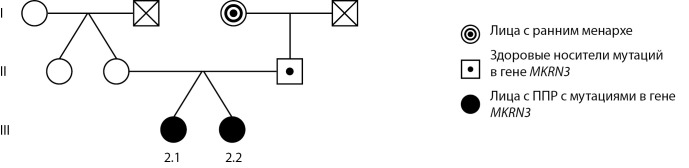
Рисунок 2. Родословная семьи 2.

**Figure:**
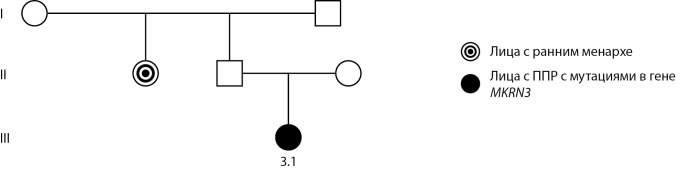
Рисунок 3. Родословная

Данные обследования приведены на момент первичного обращения. Медиана (Ме) возраста первичного обращения составила 6,4 [ 5,5; 8,3 ] года, Ме телархе — 5,3 [ 5,0; 6,3 ] года. У пациенток 1.1 и 1.3 менархе наступило в 7 лет, и к моменту обращения длительность менструаций составила 2,8 и 1,8 года. Ускорение роста и костного возраста отмечалось у всех пациентов: Ме SDS роста +2,6 [+2,4; +2,9]), Ме костного возраста 10,2 [ 9,3; 13 ]. Опережение костного возраста относительно паспортного составило 4,4 года [Ме 4,0; 4,7].

Ультразвуковое исследование выявило у всех пробандов увеличение размеров матки (относительно возрастных норм) и ее дифференцировку на тело и шейку. У пациентки 3.1 с низким базальным уровнем лютеинизирующего гормона (ЛГ) увеличение размеров дифференцированной матки и наличие эндометрия послужили дополнительным поводом проведения пробы с аналогами гонадотропин-рилизинг-гормона (ГнРГ).

По результатам гормонального исследования базальные уровни гонадотропинов соответствовали пубертатным значениям (за исключением пациентки 3.1: ЛГ 0,2 Ед/л): Ме ЛГ 2,0 Ед/л [ 1,5; 2,3]; Ме фолликулостимулирующего гормона (ФСГ) 5,4 Ед/л [ 4,5; 5,8]; Ме эстрадиола 103,4 пмоль/л [ 88,2; 116,0]. На фоне стимуляции аналогами ГнРГ Ме максимального уровня ЛГ составила 40,0 Ед/л [ 38,3; 51,5]. Пациенткам 1.1 и 1.3 проба с аналогами ГнРГ не проводилась в связи с наличием регулярных менструаций (с 7 лет).

У всех пациенток центральный генез ППР установлен на основании данных клинической картины (появление вторичных половых признаков до наступления 8 лет), ускорения костного возраста относительно паспортного (подсчет осуществлялся с использованием атласа TW20), повышения значений базального уровня ЛГ более 0,3 Ед/л и/или нарастания стимулированного уровня ЛГ на пробе с аналогами ГнРГ более 10,0 Ед/л. По результатам магнитно-резонансной томографии (МРТ) головного мозга ни в одном случае не получено данных за очаговые изменения вещества головного мозга (табл. 1).

**Table:** Таблица 1. Клинико-лабораторные данные пациентов с гонадотропинзависимым преждевременным половым развитием, обусловленным мутациями в гене MKRN3

Пациенты	1.1	1.2	1.3	2.1	2.2	3.1
Возраст пациентов при первичном обращении, лет	9,8	5,1	8,8	5,9	5,4	6,9
Пол	жен.	жен.	жен.	жен.	жен.	жен.
Возраст телархе, лет	5	4,2	5	5,5	5	6,5
Возраст менархе, лет	7	-	7	-	-	-
Стадия полового развития по Таннеру	B5P5	B3P1	B4P5	В2–3Р1	B3P1	B2P1
Рост, см	152,6	120	161,8	128	130	132
SDS роста	2,9	2,5	5,5	1,7	2,7	2,3
ИМТ, кг/м2	25,3	19,1	34,2	15,3	17,8	16,4
SDS ИМТ	2,5	2,0	3,9	-0,2	1,2	+0,4
Костный возраст, лет	14,3	9	13,9	10,2	10,2	7,8
Размеры матки (УЗИ), см	4,6×3,6×2,8шейка 2,6×1,8	4,9×2,1×1,8	4,3×3,9×2,5шейка 2,5×1,6	2,7×1,7×2,5шейка 2,7×1,4	2,4×1,6×2,5шейка 1,8×1,2	2,7×1,5×1,0шейка 1,7×1,1
Объем яичников, см3	Пр. 9,6;Лев. 10,3	Пр. 3,1;Лев. 3,7	Пр. 6,6;Лев. 5,4	Пр. 2,6Лев. 1,5	Пр. 4,7Лев. 4,2	Пр. 1,8,Лев. 1,0
ЛГ, Ед/л, 0 мин	2,6	1,51	2,3	1,1	2,0	<0,2
ФСГ, Ед/л, 0 мин	4,1	5,9	5,6	5,1	7,9	4,3
ЛГ, Ед/л, 240 мин	-	49,7	-	42,3	56,9	26,1
Эстрадиол, пмоль/л	88,2	54,9	103,4	116	213	46
Рекомендованная терапия	-	Трипторелин 3,75/28 дней	-	Трипторелин 11,25/90 дней	Трипторелин 11,25/90 дней	Трипторелин 3,75/28 дней
Рост матери/отца, см	165/178	165/178	178/182	170/177	170/177	170/182
Целевой рост, см (SD)	165 (±0,5)	165 (±0,5)	173,5 (±1,9)	167 (±0,83)	167 (±0,83)	169,5 (±1,25)

Четырем из шести пациенток рекомендована и начата терапия пролонгированными аналогами ГнРг с целью улучшения показателей конечного роста и социальной адаптации.

Учитывая возраст менархе (7 лет) и показатели костного возраста (14,3 и 13,9 года соответственно), у двух пациенток терапия аналогами ГнРГ не проводилась. Прогнозируемый конечный рост пациенток 1.1 и 1.3 (по Bayley–Pinneau) составил 157 и 165,9 см соответственно.

Молекулярно-генетический анализ проводился в лаборатории отделения наследственных эндокринопатий ФГБУ «НМИЦ эндокринологии» Минздрава России. Применялся метод таргетного секвенирования следующего поколения (NGS). Использовалась авторская панель «Гипогонадотропный гипогонадизм» (технология Ion Ampliseq™ Custom DNA Panel, Thermo Scientific, Waltham, MA, USA), содержащая праймеры для мультиплексной ПЦР и секвенирования кодирующих последовательностей следующих 30 генов: CHD7, DNMT3L, DUSP6, FGF17, FGF8, FGFR1, FLRT3, GNRH1, GNRHR, HS6ST1, IL17RD, INSL3, ANOS1, KISS1, KISS1R, LHB, NSMF, POLR3B, PROKR2, RBM28, SEMA3A, SPRY4, TACR3, WDR11, GREAT, TAC3, PROK2, NR0B1, POLR3A, MKRN3. Биоинформатическая обработка результатов секвенирования проводилась с помощью программных модулей Torrent Suite 4.2.1 (Ion Torrent, Waltham, MA, USA). Для аннотирования вариантов нуклеотидной последовательности использовался пакет программ ANNOVAR ver. 2018Apr16 [10]. Оценка патогенности вариантов нуклеотидной последовательности проводилась согласно международным и российским рекомендациям [11][12]. Нумерация кодирующей последовательности гена MKRN3 дана по референсу NM 005664.4 (http://www.ncbi.nlm.nih.gov/genbank).

У пробандов (1.1 и 1.2) и их отца, а также у двоюродной сестры (1.3) пробандов в семье 1 выявлена гетерозиготная нонсенс-мутация c.118G>T p.E40X в гене MKRN3 (рис. 4). Данное изменение раннее не описано и не встречается в базе данных gnomAD.

У пробандов (2.1 и 2.2) в семье 2 обнаружена миссенс-мутация c.343T>A p.C115S (рис. 5).

У пробанда 3.1 выявлена миссенс-мутация c.1091G>C p.C364S в гене MKRN3 (рис. 6).

**Figure:**
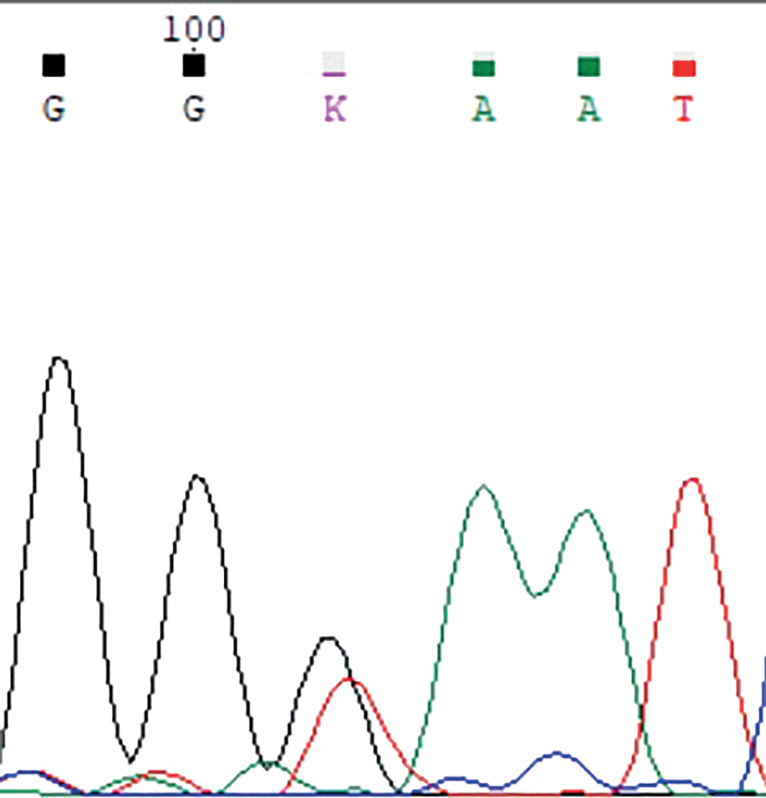
Рисунок 4. Электрофореграмма фрагмента последовательности экзона 1 гена MKRN3 у членов семьи 1: гетерозиготная трансверсия c.118G>T с заменой кодона глутаминовой кислоты (GAA) на стоп-кодон (TAA) в положении 40 (p.E40X).

**Figure:**
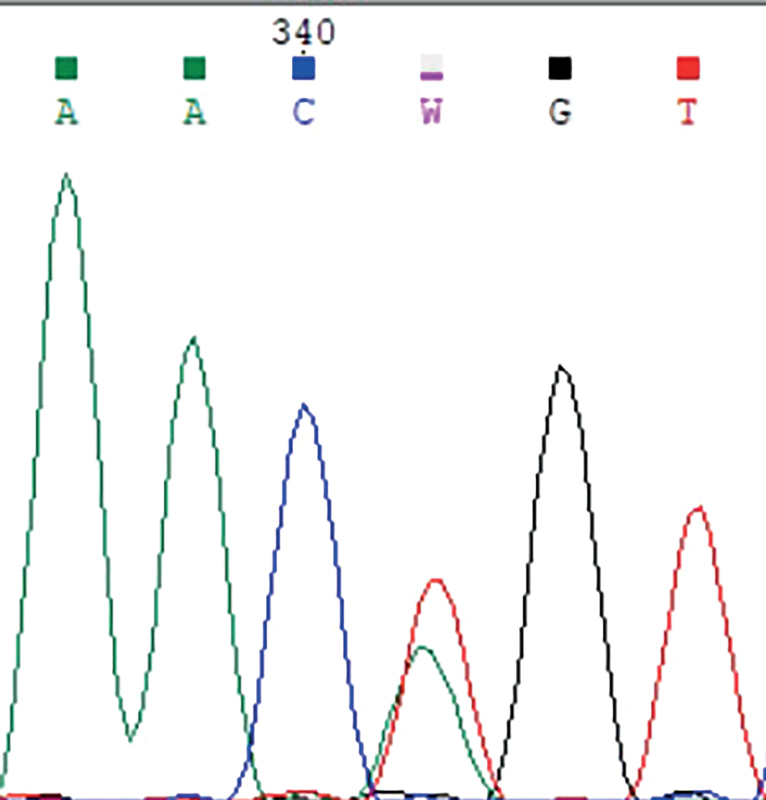
Рисунок 5. Электрофореграмма фрагмента последовательности экзона 1 гена MKRN3 у членов семьи 2: гетерозиготная трансверсия c.343T>A с заменой кодона цистеина (TGT) на серин (AGT) в положении 115 (p.C115S).

**Figure:**
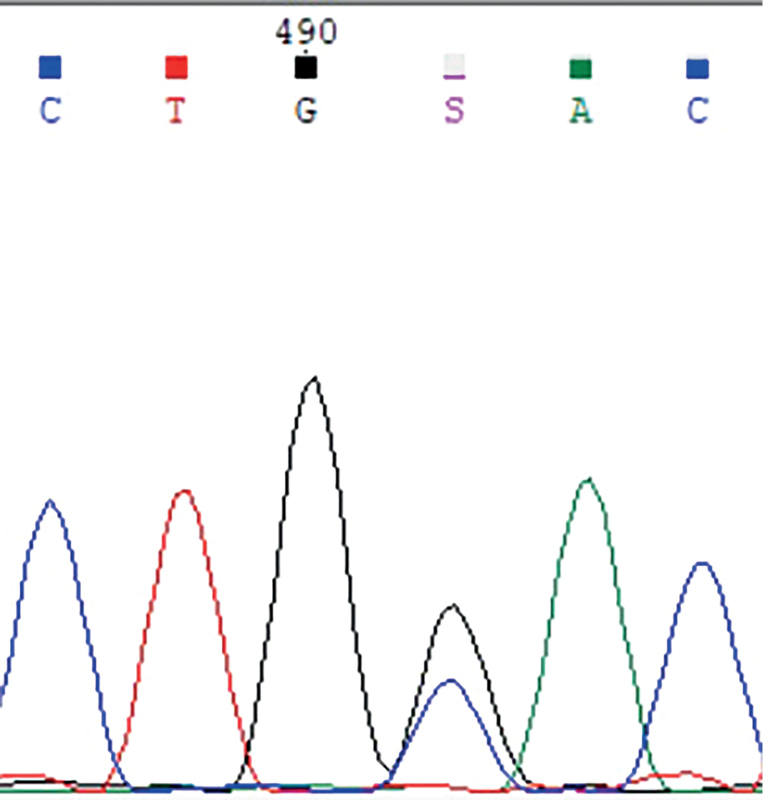
Рисунок 6. Электрофореграмма (обратная последовательность) фрагмента экзона 1 гена MKRN3 у пробанда 3.1: гетерозиготная трансверсия c.1091G>C с заменой кодона цистеина (TGC) на серин (TCC) в положении 364 (p.C364S).

Оба изменения ранее не описаны и расценены как патогенные при анализе in silico.

## ОБСУЖДЕНИЕ

Роль MKRN3 в патогенезе гонадотропинзависимого ППР впервые продемонстрирована Abreu и соавт. в 2013 г. В данном исследовании полноэкзомное секвенирование проведено 32 пациентам с центральным ППР, по результатам которого у 15 человек из 5 семей идентифицировано 5 различных нуклеотидных изменений: три инсерции со сдвигом рамки считывания, одна делеция со сдвигом рамки считывания и одна миссенс-мутация [13]. Исследования на мышиных моделях позволили сделать вывод о том, что MKRN3 играет ингибирующую роль в пубертатном периоде, а потеря его функции способствует преждевременной стимуляции секреции гонадолиберина и старту полового развития. Полученные результаты послужили началом к поиску мутаций в данном гене у пациентов с семейными формами гонадотропинзависимого ППР.

Анализ литературных данных показал, что распространенность мутаций в гене MKRN3 у пациентов с идиопатическим ППР составляет в среднем 9% [7]. К настоящему моменту описано 39 различных дефектов в гене MKRN3 у 89 пациентов (76 девочек и 13 мальчиков) из 17 стран [7].

Ген MKRN3 расположен на длинном плече хромосомы 15 (q11.2). Для данного локуса характерен геномный импринтинг, при этом материнский аллель не экспрессируется, и, соответственно заболевание развивается только в тех случаях, когда дефект унаследован от отца [14]. В когорте наших пациентов мутации в гене MKRN3 выявлены у здоровых отца и дяди в семье 1 и отца в семье 2. При этом раннее менархе отмечено у бабушек пациентов в семьях 1 и 2 и у тети по линии отца в семье 3, что послужило поводом для проведения молекулярно-генетического исследования.

MKRN3 содержит четыре типа «цинковых пальцев»: три C3H области и одну C3HC4 область, которые обеспечивают РНК-связывающую и убиквитин-лигазную активность соответственно [15]. Считается, что MKRN3 участвует в деградации белка, влияющего на пульсирующую секрецию ГнРГ, оказывая ингибирующее действие и тем самым блокируя наступление полового развития [13][16]. Точный механизм, с помощью которого дефицит MKRN3 приводит к ранней реактивации секреции ГнРГ, все еще неизвестен. В большинстве публикаций ППР связано с потерей функции в кодирующей области MKRN3, но есть указания и на единичные случаи, обусловленные дефектами в регуляторных областях гена [17][18].

В нашей когорте пациентов в гене MKRN3 выявлено 3 ранее не описанных нуклеотидных варианта: нонсенс-мутация p.E40X, миссенс-мутации c.343T>A p.C115S и c.1091G>C p.C364S, расцененные как патогенные. Мутация p.E40X приводит к образованию преждевременного стоп-кодона и, как следствие, синтезу усеченного белка, с полной потерей функции. Вариант p.C115S расположен в области «цинкового пальца» C3H, мутация p.C364S — в домене «цинкового пальца» RING C3HC4, которые связаны с убиквитин-лигазной активностью и строительством РНК. Таким образом, мутации в данных регионах должны приводить к нарушению функции белка [8]. По данным литературы известно, что домен C3HC4 является второй по частоте областью с наибольшей концентрацией нуклеотидных изменений, основную часть которых составляют миссенс-мутации [8].

Клиническая картина в случае инактивирующих мутаций в гене MKRN3, как и при иных центральных ППР, характеризуется этапным развитием вторичных половых признаков, ускорением костного возраста и пубертатным уровнем базальных и стимулированных гонадотропинов. Средний возраст начала пубертата у пациентов с мутациями в гене MKRN3, по данным объединенного исследования, составил 6,0 (3,0–7,8) года у девочек и 8,5 (5,9–9,0) года у мальчиков [19]. В группе наших пациенток возраст телархе варьировал от 4,2 до 6,9 года.

Как дополнительно показало исследование Ramos и соавт. (29 пациентов с мутациями в гене MKRN3 и 43 пациента с идиопатическим центральным ППР), сроки инициации пубертата, антропометрические данные, гормональный (базальный и стимулированные уровни гонадотропинов) и метаболический профиль, а также показатели конечного роста у пациентов с мутациями в гене MKRN3 достоверно не отличаются от таковых при идиопатическом центральном ППР [20]. Иными словами, на этапе первичного обращения крайне важен тщательный анализ клинической информации и семейного анамнеза.

У пациентов с дефектами гена MKRN3 показан адекватный ответ на терапию агонистами ГнРГ, позволяющий достичь социально-приемлемого роста [20]. Среди наших пациенток расчет прогнозируемого конечного роста, который был ниже целевого на 7,0 и 8,8 см соответственно, оказался возможен лишь у девочек (1.1 и 1.3), не получавших терапию. Данный факт демонстрирует важность своевременной диагностики семейных случаев ППР и назначения патогенетической терапии.

## ЗАКЛЮЧЕНИЕ

Впервые в Российской Федерации описаны пациенты с ППР, обусловленным мутациями в гене MKRN3, приведены их клинико-лабораторные характеристики. Идентификация данных изменений позволит в дальнейшем проводить генетическое консультирование семей, выделить группы риска по развитию ППР с последующим своевременным обследованием и назначением патогенетической терапии.
